# The clinical importance of the host anti-tumour reaction patterns in regional tumour draining lymph nodes in patients with locally advanced resectable gastric cancer: a systematic review and meta-analysis

**DOI:** 10.1007/s10120-023-01426-w

**Published:** 2023-09-30

**Authors:** Elzbieta Budginaite, Maximilian Kloft, Sander M. J. van Kuijk, Pedro A. Canao, Loes F. S. Kooreman, Alexander J. Pennings, Derek R. Magee, Henry C. Woodruff, Heike I. Grabsch

**Affiliations:** 1 Department of Pathology, GROW School for Oncology and Reproduction, Maastricht University Medical Center+, P. Debyelaan 25, 6229 HX Maastricht, The Netherlands; 2 Department of Internal Medicine, Justus-Liebig-University, Giessen, Germany; 3 Department of Clinical Epidemiology and Medical Technology Assessment, Maastricht University Medical Center+, Maastricht, The Netherlands; 4 Anatomical Pathology Department, Centro Hospitalar Universitário de São João, Porto, Portugal; 5 Faculty of Medicine of the University of Porto, Porto, Portugal; 6 Department of Surgery, GROW School for Oncology and Reproduction, Maastricht University Medical Center+, Maastricht, The Netherlands; 7 School of Computing, University of Leeds, Leeds, UK; 8 The D-Lab: Decision Support for Precision Medicine, GROW School for Oncology and Developmental Biology, Maastricht University Medical Center+, Maastricht, The Netherlands; 9 Pathology and Data Analytics, Leeds Institute of Medical Research at St James’s, University of Leeds, Leeds, UK

**Keywords:** Lymph nodes, Gastric cancer, Oesophageal cancer, Prognosis, Microarchitecture, Review

## Abstract

**Background:**

The status of regional tumour draining lymph nodes (LN) is crucial for prognostic evaluation in gastric cancer (GaC) patients. Changes in lymph node microarchitecture, such as follicular hyperplasia (FH), sinus histiocytosis (SH), or paracortical hyperplasia (PH), may be triggered by the anti-tumour immune response. However, the prognostic value of these changes in GaC patients is unclear.

**Methods:**

A systematic search in multiple databases was conducted to identify studies on the prognostic value of microarchitecture changes in regional tumour-negative and tumour-positive LNs measured on histopathological slides. Since the number of GaC publications was very limited, the search was subsequently expanded to include junctional and oesophageal cancer (OeC).

**Results:**

A total of 28 articles (17 gastric cancer, 11 oesophageal cancer) met the inclusion criteria, analyzing 26,503 lymph nodes from 3711 GaC and 1912 OeC patients. The studies described eight different types of lymph node microarchitecture changes, categorized into three patterns: hyperplasia (SH, FH, PH), cell-specific infiltration (dendritic cells, T cells, neutrophils, macrophages), and differential gene expression. Meta-analysis of five GaC studies showed a positive association between SH in tumour-negative lymph nodes and better 5-year overall survival. Pooled risk ratios for all LNs showed increased 5-year overall survival for the presence of SH and PH.

**Conclusions:**

This systematic review suggests that sinus histiocytosis and paracortical hyperplasia in regional tumour-negative lymph nodes may provide additional prognostic information for gastric and oesophageal cancer patients. Further studies are needed to better understand the lymph node reaction patterns and explore their impact of chemotherapy treatment and immunotherapy efficacy.

**Supplementary Information:**

The online version contains supplementary material available at 10.1007/s10120-023-01426-w.

## Background

Gastric cancer (GaC) is the 4th and oesophageal cancer (OeC) the 6th most common cause of cancer-related deaths worldwide, together accounting for approximately 1.3 million deaths per year [1]. Five-year overall survival (OS) of OeC and GaC patients varies between 27 and 70%, respectively, if the cancer is diagnosed at early disease stage, and is less than 10% in the locally advanced un-resectable or metastatic setting [2].

One of the most important prognostic factors in GaC and OeC patients is the regional lymph node status (N status), which is classified as N0 (no regional lymph node (LN) metastasis), N1 (1–2 LN with metastasis (positive LNs)), N2 (3–6 positive LNs), or N3 (≥ 7 positive LNs) according to Union for International Cancer Control tumour-lymph node-metastasis (UICC TNM) classification 8th ed. [3]. A high number of positive LNs as well as a high ratio of resected positive LNs (LNpos) to the total number of resected LNs are independent prognostic factors of poor survival [4]. Nevertheless, patients with the same N status can have very different survival rates. There is a clinical need to better understand the mechanisms underlying this variation and to identify potential biomarkers to personalize post-surgery treatment decisions.

The immune system plays a pivotal role as a defense mechanism against cancer. Immune evasion by cancer cells has been identified as an emerging cancer hallmark [5–7]. Several studies suggested that higher density of tumour infiltrating lymphocytes (TILs) in the primary tumour is related to a lower number of tumour-positive LNs and improved overall survival (OS) in OeC patients [8, 9]. Invasion of tumour draining LNs by cancer cells has been interpreted as ‘failed’ anti-tumour immune response in the LN in melanoma patients [10].

**Fig. 1:**
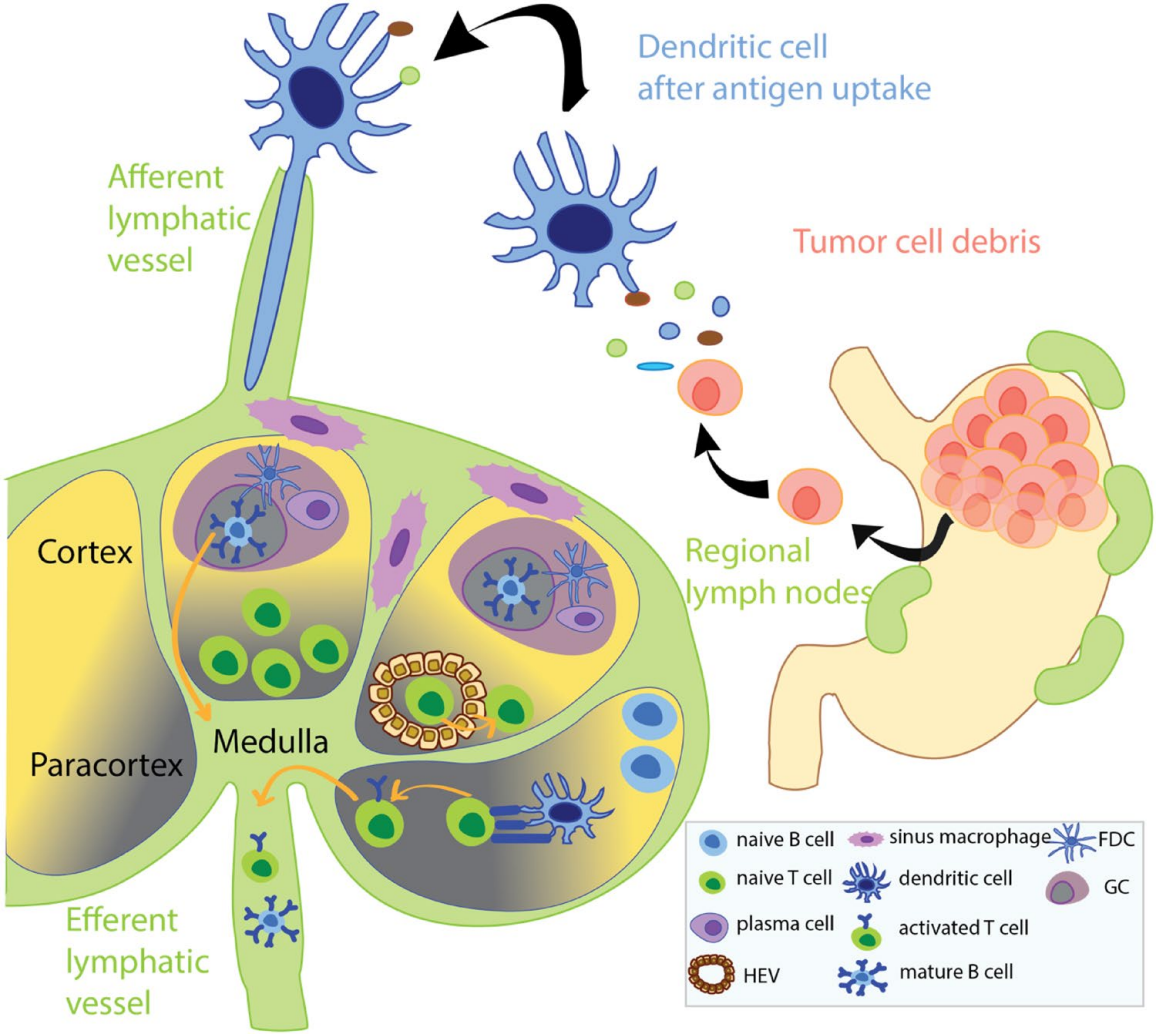
Tumour draining lymph node physiology. The tumour draining lymph fluid enters the lymph node (LN) via afferent lymphatic vessels which are connected to the capsule and the subcapsular sinus, e.g., the space between LN capsule and parenchyma. Macrophages, called histiocytes in this location, phagocytoze incoming antigens and present them to B and T lymphocytes. From the subcapsular sinus, lymph fluid drains to the cortex where immature B cells are stimulated by antigen-presenting cells (APCs) and form follicles with germinal centers (GermC). After flowing through the cortex, lymph fluid reaches first the paracortex where T cells are predominantly located and then enters the medulla where plasma cells, mature B cells and histiocytes are located. From the medulla, lymph fluid including immune cells primed to specifically attack cancer cells exits the LN via efferent lymphatic vessels into the lymphatic circulation and blood stream. *FDC* follicular dendritic cell, *HEV* high endothelial venule

Due to their unique microarchitecture, LNs provide the microenvironment necessary for an effective activation of immune cells by tumour-derived antigens [11]. A thin capsule surrounds the LN-specific parenchyma which has different microanatomical compartments, namely the cortex (outermost), paracortex (between the cortex and medulla) and medulla (Fig. 1) [12]. A steady flow of lymphocytes through all LN compartments is a prerequisite of an effective anti-tumour immune reaction [13]. Dying cancer cells are thought to be the major source of tumour-derived antigens which are captured by dendritic cells and other antigen-presenting cells (APC) close to the primary tumour site or within the LN [14, 15]. Tumour-derived antigens can either be expressed by the tumour cell or can be released by the tumour in the form of exosomes [16]. Tumour-derived antigen stimulation of LNs may lead to proliferation of anti-tumour primed T and B cells in the LN [17]. Consequently, proliferation of lymphocytes may result in (1) LN expansion and hence increasing LN size and (2) histologically visible changes of the LN microarchitecture such as follicular hyperplasia with or without formation of germinal centers (GermC) for B cell proliferation and/or paracortical hyperplasia as a result of T cell stimulation [18].

There are a number of studies in OeC and GaC highlighting the prognostic value of the immune response seen in the primary tumour [19–21]. Studies have been conducted in several different cancer types suggesting that microscopic evaluation of the LN architecture may provide important insights into the ability of an individual patient’s immune system to mount an effective anti-tumour immune response [22–26]. Despite the proposal of a classification by the World Health Organization (WHO) to standardize reporting of LN reaction patterns in 1972 [27], the scientific interest in the reactive patterns of tumour draining LN seems to have stalled after the 1980s [28, 29]. Unfortunately, the WHO LN reaction pattern classification system was never adopted into routine histopathology practice. This might be related to the fact that it requires a very detailed semi-quantitative assessment of individual LN compartments (see Supplementary Table S1).

Several studies have been published attempting to characterize the morphology of tumour-negative LN (LNneg) or LNpos in patients with OeC or GaC [24, 30, 31]. However, to the best of our knowledge, there has been no review summarizing these finding in a systematic way. We were particularly interested whether a ‘stimulated’ LN microarchitecture is related to a better prognosis in patients with OeC or GaC. LN microarchitecture assessment on routine haematoxylin/eosin (H&E) stained tissue sections might be a novel biomarker providing insight into the host anti-tumour immune response in individual patients potentially enabling personalized treatment decisions.

The objective of our study was to systematically analyze the prognostic information of LN reaction patterns in patients diagnosed with OeC or GaC, published up to 2023.

## Materials and methods

**Fig. 2:**
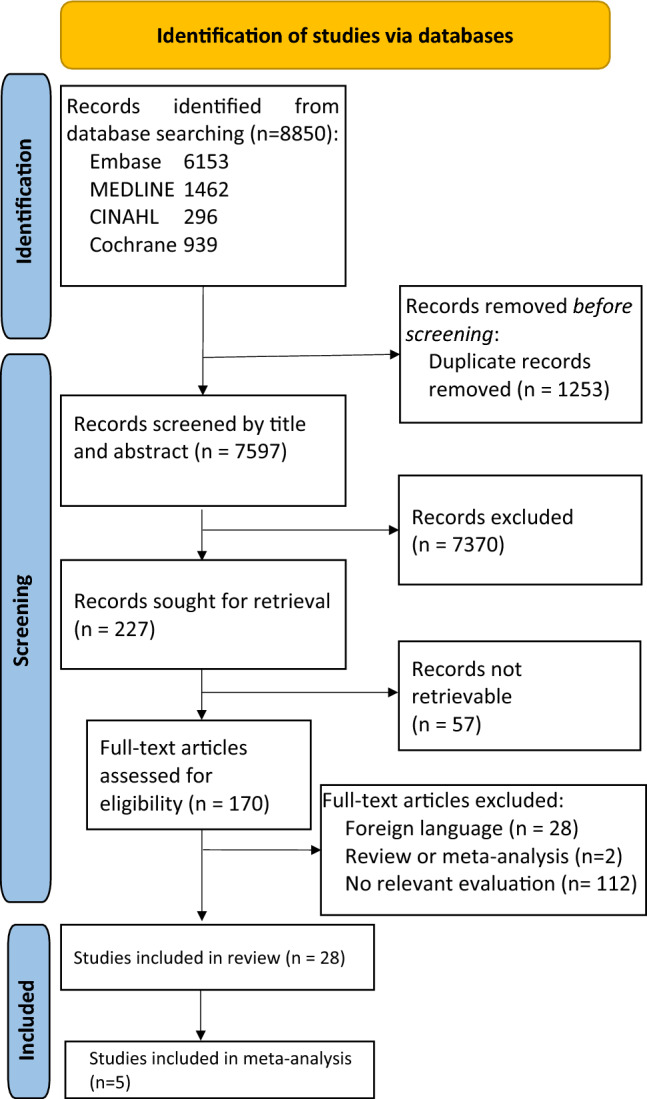
PRISM flowchart depicting meta-analysis steps

The study was conducted according to the PRISMA guidelines for systematic reviews and meta-analyses [32] and was registered at the International Prospective Register of Systematic Reviews (https://www.crd.york.ac.uk/PROSPERO/, No. CRD42021260650) (Fig. 2).

### Search strategy

A systematic search of the literature was performed using the Embase, MEDLINE, CINAHL, and Cochrane databases. Relevant studies published before April 2023 were collected using Medical Subject Headings (MeSH) terms as mentioned in Supplementary Table S2. Reference lists from relevant papers were screened to identify further relevant original publications.

### Study selection

As the initial search showed a relatively low yield when only including gastric cancer, we extended the search to include junctional and oesophageal cancer. The following criteria were used to identify studies eligible for inclusion in our analyses: (1) patients diagnosed with gastric, junctional or oesophageal cancer treated by surgical resection including lymph node dissection; (2) histological examination of morphological changes in the lymph node microarchitecture using H&E staining or other staining procedures; (3) acceptable study design: cohort studies, randomized controlled trials and case control studies; (4) full-text available in English, German, Dutch, Czech, or Japanese. Studies reporting results from case reports, cell culture-based studies, animal studies, diagnosis based on cytology, and letters to editors containing no additional information were excluded.

Study selection was performed independently by two authors (MK and EB) considering the pre-defined inclusion and exclusion criteria. The eligibility of the papers was evaluated by initial screening of title and abstract, followed by evaluation of the full text. Inter-evaluator disagreement regarding eligibility of a study to be included or not was resolved by discussion with a third researcher (HIG).

### Data extraction

Initially, the following data items were collected: author, year of publication, country of the researcher’s affiliation, cancer type, histological type, number of patients, patient demographics (age, sex), number of lymph nodes (LNs) analyzed per patient including number of metastatic and non-metastatic LNs, criteria used by investigators to identify LN suitable for analyses, LN reactive pattern(s) analyzed in the study, histological stain used, evaluation method, result measures (quantitative versus qualitative), treatment groups if applicable, survival time, other prognostic positive/negative parameters and primary, and secondary endpoints reported in the study.

The main research outcome of interest was the relationship between LN microarchitecture feature and survival in patients with GaC or OeC treated with surgery with or without neoadjuvant or adjuvant treatment. Alternative study outcomes such as association of the LN reaction pattern with clinicopathological variables other than survival were also considered. To account for potential risk of bias in the studies, the Quality in Prognostic Studies (QUIPS) tool was applied to selected studies [33].

### Statistical analyses

The meta-analysis was performed using R packages metafor (version 3.8–1) and ggforestplot (version 0.1.0) for visualization. In order to compare survival between patients who exhibited a reactive LN pattern against those without, pooled risk ratios (RR) and 95% confidence intervals were calculated using the DerSimonian and Laird algorithm [34]. The clinical heterogeneity of the studies was used to determine random or fixed model suitability, accounting for preoperative treatment, patient geography, mean age, sample size, and male/female ratio of the participants in the included studies.

## Results

### Database search and article selection

Following the database search, 7597 unique records were retrieved for subsequent title- and abstract-based screening. The selection process is outlined in Fig. 1. A total of 7370 records were excluded as they did not fulfill the predefined inclusion criteria. From the remaining 227 articles, the full text was available for 170 studies. After reading the full text, 142 of the 170 studies did not meet the inclusion criteria leaving 28 studies for final analyses within this systematic review. Five of the 28 studies provided sufficient details on number of patients with reactive LN patterns and patient survival to be included in the meta-analysis. As individual patient data were not available from these studies (all published before 1992), the overall survival data was obtained from publication text, tables, or calculated from published survival curves. Observations reported by Kuriya et al. could not be included into the meta-analysis, as we were unable to calculate the required values from survival curves plotted in the paper [35]. The risk ratios were assessed using a random-effects model, since the study pool was not homogeneous with respect to the age, sex distribution of the investigated patients as well as the geographical location, as described in Table 1. Based on the QUIPS risk of bias score evaluation, 18 (64%) articles were assigned medium risk of bias, leaving the remaining 10 articles (36%) with low risk scores (see Supplementary Table S3).

**Table 1:** Relevant studies on lymph node microarchitecture remodeling in upper gastrointestinal cancer patients

Author (Year)	Country	Type of cancer	Age mean/range	Sample size/ Male fraction (%)	Number of investigated LNs (^a^no data, one per patient inferred)	Neoadjuvant preoperative treatment	Type of staining	OS time	Main finding	QUIPS risk of bias score
**I Category: LN compartment specific hyperplasias**										
Black (1971) [31]	USA	GaC	–/–	592/65.71	592^a^	None	H&E	5-year	FH associated with favorable outcomes	Low
Kodama (1976) [24]	Japan	GaC	–/–	141/–	2916	None	H&E	5-year	PH, FH and PH FH combined associated with favorable outcomes, SH insignificant	Medium
Syrjanen (1977) [44]	Finland	GaC	–/–	138/–	295	None	H&E	5-year	PH associated with favorable outcomes	Medium
Kuriya (1980) [35]	Japan	OeC	63/47–71	21/–	152	not specified	H&E	3 and 5-year	SH associated with favorable outcomes for 1–5-year survival, FH insignificant	Medium
Kojima (1980) [45]	Japan	GaC	51.5/32–68	32/–	70	not specified	H&E, methyl green pylonin stain	–	PH and FH associated to immune responsiveness	Medium
Oka (1981) [30]	Japan	OeC	–/–	66/–	66^a^	radiotherapy	H&E	1 year	SH associated with favorable outcomes in both radio and nonradio pretreatment groups, FH – only in no pretreated group	Medium
Riegrova (1981) [38]	Czech Republic	GaC	–/36–80	80/60	80^a^	None	H&E	5-year	PH and SH favorable prognostic indicators	Medium
Okamura (1983) [42]	Japan	GaC	–/–	700/–	3330	not specified	H&E, methyl green pylonin stain	5-year	High rate of PH and FH in lymphoid stroma group. LN reaction pattern not related to survival	Medium
Bedikian (1984) [39]	USA	GaC	62/25–100	783/66.67	783^a^	5-FU control group	H&E	5-year	SH associated with favorable outcomes	Low
Eriguchi (1984) [41]	Japan	GaC	–/–	33/–	33^a^	BCG + chemoimmuntherapy	H&E	2 year	Group with BCG administration better OS, SH not related to OS	Low
Oka (1992) [37]	Japan	GaC	–/32–81	102/61.77	3267	None	H&E	5-year	SH associated with favorable outcomes, FH insignificant	Medium
Lu (1994) [40]	China	OeC	–/19–72	1033/70.38	1033^a^	not specified	H&E	10 year	FH more frequent in older age group, SH—no significant differnece	Low
Kloft (2022) [43]	Netherlands	OeC	62.5/30–83.1	93/–	93	chemo + surgery	H&E	5-year	Large LNneg have more germinal centers, less lymphocytes	Medium
**II Category: Cell-type infiltration**										
Ikeguchi (1998) [46]	Japan	OeC	64/–	88/87.5	88^a^	None	H&E/IHC	3 year	S100 + DCs associated with favorable outcomes	Medium
Ishigami (2003) [49]	Japan	GaC	60/31–86	27/62.96	35	None	H&E/IHC	–	Immune cell spatial infiltrate signatures	Medium
Lee (2011) [50]	Korea	GaC	–/–	64/54.69	64^a^	None	H&E/IHC	–	FoxP3 + Tregs associated with unfavorable outcomes	Medium
Kashimura (2012) [47]	Japan	GaC	66.9/–	123/72.36	123^a^	None	H&E/IHC	10 year	CD83 + DCs associated with favorable outcomes, FoxP3 + Tregs – with unfavorable	Low
Tokumoto (2014) [55]	Japan	GaC	–/–	52/–	364	None	H&E/IHC	5-year	TANs associated with unfavorable outcomes	Medium
Go (2016) [53]	Japan	GaC	–/–	49/–	1448	not specified	H&E/IHC	–	CD163 + TAMs associated with unfavorable outcomes	Medium
Hiramatsu (2018) [54]	Japan	GaC	–/–	120/72.5	480	not specified	H&E/IHC	5-year	TANs associated with unfavorable outcomes	Medium
Takeya (2018) [51]	Japan	OeC	65/–	182/87.91	182^a^	chemo + chemoradiotherapy	IHC	10 year	CD169 + LyMSs corelates with TIL count in primary tumour	Medium
Takeya (2019) [52]	Japan	OeC	–/–	182/87.91	182^a^	not specified	H&E/IHC	10 year	Gene expression heterogeineity in CD169 + LySMs	Low
Donlon (2021) [75]	Ireland	OeC	–	–	6	Chemo and chemoradio therapies	None, flow cytometry	–	Higher expression of inhibitory immune checkpoints in TME compared to TDLNs	Medium
Kumamoto (2021) [76]	Japan	GaC	–	284/–	294	Not specified	H&E/IHC	5	CD169 + LySMs positively correlated to CD8 + lymphocytes in primary tumour and associated with favorable CSS	Low
Itai (2021) [77]	Japan	GaC	71.9/24–92	391/69.3	10,149	None	H&E/IHC	3	Sarcoid reaction associated with better prognosis for 3-year OS and RFS, CD68 + macrophages present in sarcoid-positive LNs	Low
**III Category: Gene Expression pattern**										
O'Sullivan(1996) [58]	Ireland	OeC	–/41–88	23/47.83	138	None	None	5-year	Immunosuppressed TDLNs in ESCC compared to adenocarcinoma control TDLNs	Low
Otto (2014) [56]	Germany	OeC	–/–	22/–	44	None	H&E/IHC	2 year	Downregulated DKK1 (Wnt inhibitor) expression in premetastatic TDLNs	Medium
Jia (2015) [57]	China	OeC	54/29–73	196/67.35	196^a^	None	H&E/IHC	10 year	Low Bin1 and high IDO expression as independent prognostic negative factor	Low

*OS* overall survival, *QUIPS* Quality in prognostic studies, *GaC* gastric cancer, *OeC* oesophageal cancer, *CRC* colorectal cancer, *GEJC* gastro-oesophageal junction cancer, *FU* fluorouracil, *BCG* Bacillus Calmette–Guérin, *H&E* haematoxylin and eosin, *IHC* immunohistochemistry, *PAS* periodic acid-Schiff, *SH* sinus histiocytosis, *PH* paracortical hyperplasia, *FH* follicular hyperplasia, *TAN* tumour associated neutrophils, *LySM* lymph node sinus macrophages, *TAM* tumour associated macrophage, *DC* dendritic cell, *TGF* tumour growth factor, *IDO* indoleamine 2, 3-dioxigenase, *DKK1* Dickkopf-related protein 1, *TDLN* tumour draining lymph node, *CSS* cancer-specific survival, *RFS* recurrence-free survival

^a^No data, one per patient inferred

The studies included in this review consisted of 17 studies in gastric cancer (GaC) patients and 11 studies in oesophageal cancer (OeC) patients including 5685 patients and 26,503 investigated LNs. On average, the LN reaction pattern was investigated in 4.66 LNs per patient, including tumour-positive and tumour-negative LN. Thirteen studies reported histopathological findings based on H&E-stained LNs, 16 studies used immunohistochemistry (IHC) or gene expression patterns (see Table 1). Studies investigating gene expression or specific immune cell type infiltration patterns were more recently published compared to studies reporting on LN compartment hyperplasia based on H&E-stained sections. Studies originated from several different geographical locations, see Table 1.

Overall, the 28 studies could be grouped into three different methodological approaches: (1) Thirteen (46%) studies investigated LN compartment specific reactions in GaC and OeC, namely sinus histiocytosis, paracortical hyperplasia, and follicular hyperplasia (SH, PH, FH, respectively) in H&E-stained tissue sections (see supplement Figure S1). Five of these studies contained detailed information on the relationship between LN reaction pattern and overall survival and were therefore deemed eligible for meta-analysis. (2) Twelve (43%) studies used immunohistochemistry to characterize specific immune cell distribution patterns in LNs (Table 1). (3) Three (11%) studies investigated RNA expression to identify differential gene expression signatures in LNs.

### Clinical significance of hyperplastic lymph node compartments

Thirteen studies measured volumetric changes in regional LNs of GaC and OeC patients, all of which were based on H&E staining. Although the studies shared the same LN reaction pattern terminology, namely sinus histiocytosis, follicular hyperplasia, and paracortical hyperplasia, the morphological evaluation itself was inconsistent between studies due to the use of different grading systems. The purpose of these grading systems was to semi-quantify the findings of the LN reaction patterns. Six different grading systems were used for sinus histiocytosis, four different grading systems for follicular hyperplasia, and three different grading systems for paracortical hyperplasia, see Table 2. It is noteworthy that the WHO LN reaction pattern classification by Cottier in 1972 [27] included evaluation of the LN medulla for medullary plasmacytosis, which was not investigated in any of the included gastro-oesophageal cancer studies.

**Table 2:** Grading systems proposed for lymph node reactive microarchitecture evaluation

Reaction	Author	Grading criteria	Studies adopting the grading system
Sinus histiocytosis	Black (1958) [36]	Grade 1: < 2 histiocytes across the sinus Grade 2: 2–4 histiocytes Grade 3: 5–7 histiocytes Grade 4: ≥ 8 histiocytes	Kuriya (1980) [35] Black (1971) [31] Eriguchi (1984) [41] Lu (1994) [40]
	Fujimaki (Japanese study)	Manuscript not available	Eriguchi (1984) [41]
	Cottier (1972) [27]	+ / + + / + + + grading system based on histiocyte density in sinuses	Kodama (1976) [24] Okamura (1983) [42]
	Tsakraklides (1973) [78]	Lymphocyte predominance coupled with SH as one reactive pattern	Kojima (1980) [45]
	Oka (1981) [30]	Grade 1: 0 area of LN Grade 2: less than ¼ LN area Grade 3: > ¼ ≤ ½ LN area Grade 4: > ½ LN area	Oka (1981) [30] Oka (1992) [37]
	Bedikian (1984) [39]	Binary evaluation: SH present or absent	Bedikian (1984) [39]
Follicular hyperplasia	Black (1971) [31]	Binary: presence of GermCs in the majority of the cortex	Kuriya (1980) [35] Black (1971) [31] Lu (1994) [40]
	Cottier (1972) [27]	+ / + + / + + + grading system based on GermC size, number, mitotic activity	Okamura (1983) [42]
	Tsakraklides (1973) [78]	GermCs with large lymphoid cells showing mitotic figures	Kojima (1980) [45]
	Oka (1981) [30]	Grade 1: rare follicles Grade 2: located in cortex Grade 3: expanded to medulla Grade 4: in all LN areas	Oka (1981) [30]
Paracortical hyperplasia	Cottier (1972) [27]	+ / + + / + + + grading system based on size, small/ medium-sized lymphocyte counts, large lymphocytes, histiocytes	Okamura (1983) [42]
	Syrjanen (1977) [44]	Small lymphocyte density, HEV endothelial morphology, intraluminal content	Syrjanen (1977) [44]
	Jansa and Pastrnák (1981) [79]	Manuscript not available	Riegrova (1981) [38]
Medullary plasmacytosis	Cottier (1972) [27]	Size, cord width, large lymphoid, plasma cell, lymphocyte counts	–

### Sinus histiocytosis

We identified ten studies investigating sinus histiocytosis (SH) in GaC and OeC using multiple different grading systems. Black et al. proposed a semi-quantitative four stage grading for SH evaluation, based on manually counting of the number of histiocytes across the sinus [36] (see Table 2). Oka et al. measured the proportion of the lymph node area covered by sinuses [30].

Five studies investigating SH reported a significant association between the presence of SH and favorable outcomes. Oka reported favorable outcomes with increased incidence of SH irrespective of preoperative radiotherapy treatment in OeC patients and GaC patients [30, 37]. Presence of SH in regional LNs was also related to better survival in GaC by Riegrova and Bedikian [38, 39]. Kuriya et al. associated SH with better prognosis in oesophageal squamous carcinoma patients using the SH grading system proposed by Black et al. [35]. Black et al. reported a relationship of SH with improved OS which did not reach significance probably due to low incidence of SH in their GaC study cohort (LNs from only 30 (5%) out of 592 patients showed SH) [31]. Similarly, Lu et al. used a qualitative adaptation of Black’s SH grading and observed no association of SH with clinical outcome in a large oesophageal squamous cell carcinoma patient cohort (186 (18%) out of 1,033 patients with SH positive LN) [40]. Following Black’s SH evaluation strategy, Eriguchi observed higher SH incidence in LNs from Bacillus Calmette–Guérin (BCG)-treated GaC patients compared to control [41].

Okamura and Kodama have used the SH grading system (none versus slight versus obvious) proposed by Cottier [27]. Both studies reported a relatively low SH incidence in GaC cohorts (21 (15%) out of 141 patients in Kodama’s study, 26 (14%) out of 189 patients in Okamura’s) and no relationship between presence of SH and survival [24, 42].

### Follicular hyperplasia

A potential clinical value of follicular hyperplasia (FH) was suggested by seven studies both in GaC and OeC patients, for details, see Table 1. Similar to reports on SH, the lack of an universally accepted grading system is reflected by a variety of FH grading approaches (Table 2). Black et al. proposed a binary classifier based on the presence or absence of secondary follicles in the LN cortex defined as a germinal center (GermC) surrounded by a mantle zone [31], whereas Oka et al. proposed to account for varying extent of FH in the LN by distinguishing GermC presence in the cortex versus medullary area [30]. LNs exhibiting both, FH and paracortical hyperplasia (PH) have been associated with a longer 5-year survival in a GaC study by Kodama et al. Okamura’s results suggest that presence of concurrent PH and FH is related to an increased number of tumour-infiltrating lymphocytes in primary GaC [24, 42]. Black et al. found an association between presence of FH and favorable survival [31].

A relationship between LN area occupied by GermCs in tumour-negative LNs of OeC patients and survival was recently confirmed by Kloft et al. [43]. In the same study, it was suggested that LN compartment hyperplasias might contribute to an increase in LNneg diameter, which was found to be an independent favorable prognostic marker.

### Paracortical hyperplasia

The expansion of the paracortical area, termed paracortical hyperplasia (PH), is histologically visible as an increase in lymphocytes located between the cortex and medulla of the LN. The presence of high endothelial venules (HEVs) in this region has been proposed as a criterion for PH by some investigators [27, 44]. In the study by Syrjanen et al., PH together with high cuboidal shape of endothelial cells in HEV located in the paracortex was associated with better overall survival (OS) and lower number of regional LN metastasis in GaC patients. Findings of an experimental, translational approach by Kojima et al. revealed a higher lymphocyte reactivity upon antigen stimulation in cell cultures if lymphocytes were derived from GaC patients LNs exhibiting paracortical activation [44, 45]. Likewise, Riegrova et al. found that increased PH was associated with increased survival in GaC patients [38].

### The immune cell landscape of reactive lymph nodes

The immune cell signature of tumour draining LNs was investigated by immunohistochemistry (IHC) in twelve studies. The density of dendritic cells (DCs), known for their antigen-presenting capacity and T cell response induction, was measured as IHC-positive cell density per area or as total cell count and shown to be associated with lower number of LN metastases and lower grade of tumour differentiation in regional LNs in GaC and OeC studies, see Supplementary Tables S4 and S5 [46–48]. No significant differences in the extent of DC density were observed between tumour-positive and tumour-negative sentinel LNs in GaC studies by Ishigami et al. and Lee et al., see Supplementary Table S4 [48, 49]. Ishigami et al. reported no significant cell density differences in natural killer (NK) cells between sentinel and non-sentinel LNs in GaC neither in tumour-negative nor in tumour-positive LNs [49]. A significant association was reported between the presence of LN metastasis and the density of FOXP3+ T-regulatory cells (Tregs) in the LNs of GaC patient by Lee et al. [50]. CD169+ macrophages in LNs are functionally dependent on DCs, and higher numbers of CD169+ macrophages in LNs have been associated with favorable outcome in OeC patients [51, 52]. Takeya showed an association between LN resident CD169+ sinus macrophages (LySMs) and higher tumour infiltrating lymphocyte (TIL) counts in patients after neoadjuvant therapy, suggesting host immune reactivity induced by cancer cell phagocytosis by LySMs [51]. Besides cancer cells, immune-suppressive macrophages from the tumour microenvironment (TME) are also well-established modulators of LN reactions. The studies by Go et al., Hiramatsu et al., and Tokumoto et al. showed that the presence of tumour associated CD163+ macrophages (TAMs) and CD15+ neutrophils (TANs) in tumour draining LNs was related to poor survival in GaC patients [53–55].

### Gene expression signatures in tumour-positive and tumour-negative LNs

Otto et al. has reported a significant down-regulation of DKK1 in the subcapsular sinuses and in  endothelial cells of pre-metastatic regional LNs in OeC patients [56]. Overexpression of the IDO gene, coupled with downregulation of tumour suppressing BIN1 in LNs was associated with poor prognosis in OeC patients in a study by Jia et al. [57]. O’Sullivan et al. reported a significant suppression of the proliferation of LN-derived lymphocytes in oesophageal squamous cell carcinoma compared to oesophageal adenocarcinoma cases, suggesting cancer subtype-specific immune response mechanisms in LNs [58].

### Meta-analysis corroborates clinically significant LN reaction patterns

Five GaC studies with well-defined LN reactive patterns and sufficiently large number of patients were used for meta-analysis (Black et al. 1971, Kodama et al. 1976, Oka et al. 1981, Riegrova et al. 1982, Oka et al. 1992) [24, 30, 31, 37].

**Fig. 3:**
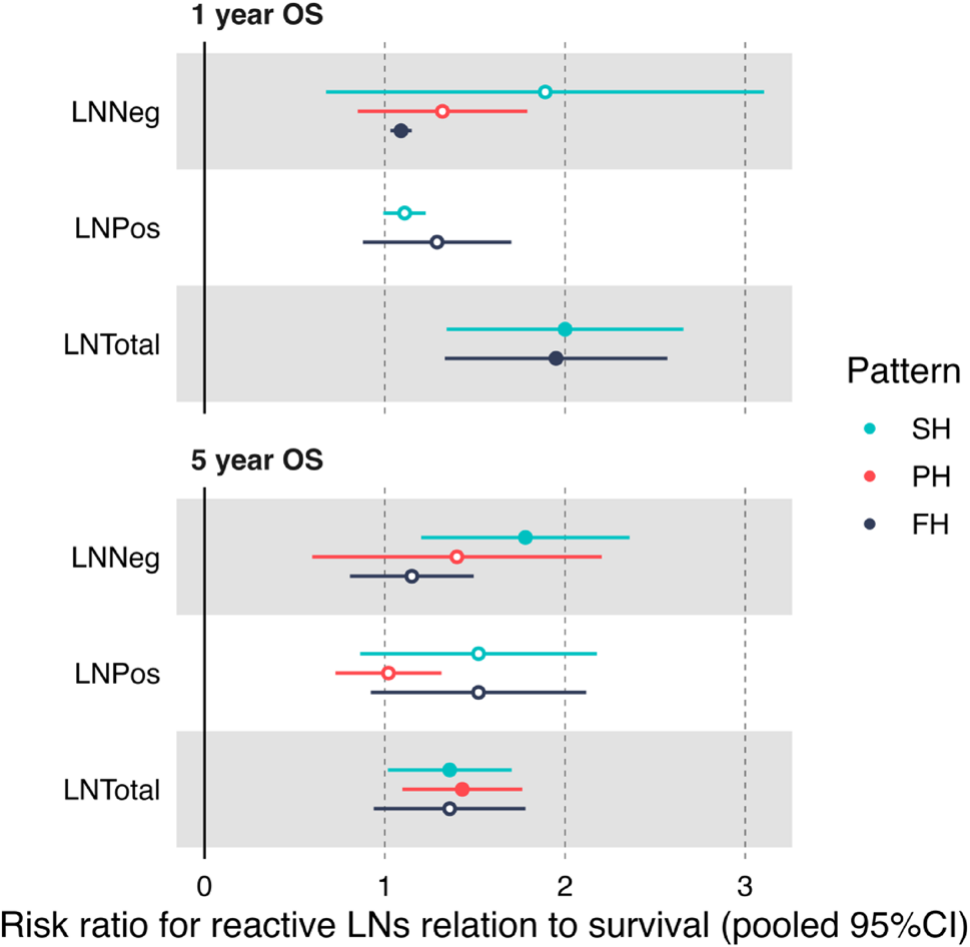
Forest plot of pooled risk ratios with 95% confidence interval (CI) depicting the risk of surviving 1 or 5 years for patients with reactive lymph nodes compared to patients with non-reactive lymph nodes. A favorable 5-year overall survival outcome could be shown for patients with SH and PH in the investigated LN pooling LNpos and LNneg. SH was also significantly associated with survival when investigating LNneg alone. In the 1-year OS analysis, the pooled analysis of LNneg and LNpos indicated that SH and FH were both  associated with a favorable outcome. *LN* Lymph node, *LNNeg* non-metastatic lymph nodes, *LNPos* metastatic lymph nodes, *LNTotal* all lymph nodes irrespective of metastatic state, *SH* sinus histiocytosis, *PH* paracortical hyperplasia, *FH* follicular hyperplasia.  Note that the forest plot items with color-filled dots represent statistically significant observations in the meta-analysis

Our meta-analysis showed a statistically significant association between the presence of SH and favorable outcome with pooled risk ratios (RR) and 95% confidence intervals (CI) of 2.73 [1.23–6.05] and 1.36 [1.01–1.82] for 1- and 5-year survival, respectively, as well as 1.78 [1.19–2.66] for 5-year OS when analyzing results from tumour-negative LNs (Fig. 3). Forest plots showing the study-specific weights and *p* values are provided in Supplementary Figure S2A-O. Furthermore, the meta-analysis showed a significant association between the presence of FH and favorable 1-year OS with RR [95% CI] of 1.09 [1.03–1.16] and 1.85 [1.32–2.59] in tumour-negative and all investigated LNs, respectively. A significant association with favorable 5-year OS was detected when pooling results from tumour-negative and tumour-positive LNs exhibiting PH with RR [95% CI] of 1.43 [1.09–1.88]. The studies exhibited high heterogeneity with respect to age, sex distribution, and geography of selected patients, rendering the data eligible for random-effects model.

To compare the incidence of LN reactive patterns between tumour-negative and tumour-positive LNs, the incidence of reactive LN patterns was pooled from all available GaC studies. Our analysis showed that tumour-negative regional LNs had a twice higher incidence of reactive pattern compared to tumour-positive regional LNs (see Table 3). Although a similar analysis was not feasible in OeC studies due to missing data per LN metastatic state, the pooled SH and FH frequencies from three OeC studies showed FH in 54.9% LNs compared to SH in 19.6% LNs.

**Table 3:** Incidence of reactive lymph nodes (LNs) in upper GI cancer patients retrieved from the reviewed studies

Reaction pattern	Gastric cancer				Oesophageal cancer	
	Tumour-negative LNs		Tumour-positive LNs		All LNs	
	Total	% Reactive	Total	% Reactive	Total	% Reactive
SH	154	45.4	358	23.18	1094	19.6
PH	448	38.6	353	16.4	–	–
FH	261	42.4	482	25.3	1084	54.9

*SH* sinus histiocytosis, *FH* folliclar hyperplasia, *PH* paracortical hyperplasia

## Discussion

This is the first systematic review of the existing literature on the microarchitecture of regional tumour draining lymph nodes (LNs) in oesophageal and gastric cancer.

The structural rearrangements in regional tumour draining LNs in cancer patients have been recognized as a potentially clinically relevant readout of the host’s anti-tumour response in various cancer types in the past [59]. Yet, the lack of objectively quantifiable histomorphological metrics hinders the applicability of assessing LN microarchitecture in daily clinical practice. In this systematic review, we aimed to summarize the existing evidence regarding the morphological reaction patterns in LNs of patients with oesophageal (OeC) or gastric cancer (GaC). We identified numerous alternative protocols for LN reactivity measurement making comparisons between results from different studies difficult.

In the current review, we have found that sinus histiocytosis (SH) was the most frequently studied reaction pattern and SH presence was found to be related to improved survival. The reaction patterns follicular hyperplasia (FH) and paracortical hyperplasia (PH) were less frequently studied but showed to be of prognostic value in six studies.

Our systematic analysis of the literature seems to indicate that the presence of any reaction pattern in regional tumour draining LNs is beneficial for patient survival. Interestingly, some studies suggested that having more than one reactive pattern in the same LN was associated with better prognosis than showing a single reaction pattern only. However, from the included studies, it is not entirely clear what the interaction between the reaction patterns is.

Furthermore, as the evaluation on LN specimens is always a measure at a certain time point, it is currently unclear whether LN reaction patterns happen in a particular temporal order or whether LN morphology is undergoing continuous remodeling as suggested by Drayton et al. [60].

Sinus histiocytosis has been interpreted as the primary, yet immature response of the LN to tumour-derived antigens [1]. Sinus histiocytosis is thought to precede secondary responses like follicular hyperplasia or paracortical hyperplasia [61]. However, the extent of sinus histiocytosis is difficult to measure due to the varying diameter of sinuses across the transversal plane of the LN. A subjective method of evaluating the area of sinus histiocytosis has been proposed by Cutler and Black [31, 62]. However, the authors also showed that SH evaluation is prone to inter-observer variation even after repeated training and consensus attempts [31]. The area ratio-based SH evaluation suggested by Oka et al. [30] leaves less room for interpretation, yet this method is still prone to errors as histiocyte-devoid dilated areas could be included by mistake. Interestingly, the reported incidence of SH in GaC LNs seems to be lower compared to that reported in breast cancer LNs, which might be related to a different extent of antigen stimulation between axillary and mesenteric lymph nodes, with the latter being constantly exposed to antigens from the gastrointestinal tract [27, 31].

Follicular hyperplasia in tumour draining LN has been associated with favorable prognosis in breast cancer [63]. Back in the 1970s, Cottier emphasized that a prolonged antigen stimulation can lead to germinal center (GermC) expansion to the medullary region of LN [27]. More recently, Grigoriadis et al. proposed a multi-factorial FH grading system considering spatial arrangement, number of GermC (grade included absent to numerous), and GermC size in breast cancer patients [64]. Using this grading system, Alberts et al. observed a favorable association with survival for the presence of multiple small cortex-located GermC in tumour-negative LNs compared to few large GermC extending to the medulla in breast cancer [65]. Lymphoid follicles with GermCs forming so-called tertiary lymphoid tissues (TLS) can be seen outside of LNs within tumour and normal tissue. Presence of TLS has been associated with longer overall survival, lower recurrence rate, and decreased chemotherapy resistance in GaC patients [66]. Besides the spatial distribution within the LN, the shape of the GermC might be related to patient prognosis. Unger et al. employed elementary geometrics to quantify ‘roundness’ of GermC in order to account for GermC shape deviation from a perfect circle. GermC shape irregularities seem to be associated with immune disorders in patients with common variable immunodeficiency [67].

Activated host immunity can also manifest itself as accumulation of lymphocytes in the LN paracortex with occasional expansion to the medulla, historically referred to as paracortical hyperplasia [27]. One typical characteristic of PH is the presence of high endothelial venules (HEV). These enable lymphocyte migration in and out of the LN, thus enhancing immune cell interaction to execute immune responses originating in the LN.

Unfortunately, the protocols used for the studies were inconsistent in including HEVs residing in paracortical area of LNs and assessing their morphological alterations upon paracortical stimulation. Based on our literature search, the study by Syrjaen et al. was the only one in GaC to address the shape of endothelial cells of HEVs and the presence of intraluminal lymphocytes. The authors suggested that there is a prognostic difference between HEV with cuboidal endothelial cells as a sign of antigen stimulation and HEVs with thin and flattened endothelial cells [44]. Syrjaen’s conclusions were supported by Farnsworth’s studies showing a transformation of cuboidal endothelial cells to flat, thin-walled endothelial cells HEVs in tumour-positive and tumour-negative LN by tumour-derived signal in murine models of breast cancer [68]. This could indicate that LN remodeling takes place resulting in the formation of the so-called pre-metastatic niche. These purely morphological observations are supported by Otto et al’s observation that the expression of DKK1 gene, an angiogenesis suppressor [68], appears to be downregulated in pre-metastatic LNs of OeC, whereby endothelial cells seem to be the main cell type expressing DKK1 in LN [56].

Based on our systematic review, there seems to be important prognostic information in the LN reaction pattern, although the evidence is not entirely consistent. This is most likely related to the fact that studies included in this review did not use a standardized evaluation method for LN reaction patterns. Cottier et al. proposed to account for specific immune cells in LN sinuses such as histiocytes, lymphocytes, large lymphoid cells, neutrophils, eosinophils, and mast cells separately [27]. However, the grading of each component is based on semi-quantitative three-category scale, that could be susceptible to inter-observer variation [27].

The contemporary histopathology protocols for upper gastrointestinal cancer reporting include the recommended minimal number of resected LNs, pN staging (number of positive LNs per resection specimen), recommendations on the dissection procedure (section perpendicular to the longest axis), number of sections per LN (single midsection for small LNs and multiple sections along the longest axis for larger ones), and gross anatomical LN description (weight, site, size) [69–72]. Pathological abnormalities visible in the LN section, including tissue discoloration, unusual consistency, and presence of necrotic or nodular areas should also be reported in particular in the context of lymphoma screening procedures [73]. However, modern guidelines lack a detailed template for the description of the tumour-negative LN architecture, hence, Cottier’s pathology guidelines from 1972 are currently the only guidelines to standardize LN reactive morphology reporting [27].

Based on the current literature and guidelines [69–72], we propose a set of recommendations that might help standardizing the reporting of reactive patterns in tumour draining LNs (Table 4). Implementing these recommendations in future morphologic LN assessment could aid to accumulate precise information on the underlying immune biology of anti-tumour defense mechanisms within LN of cancer patients. For example, spatial analysis of LN components might provide insights into immune cell interactions within the LN influencing the degree of immune cell activation. Additionally, retrieving information about the morphology, location and number of HEVs in the LN may provide clinically relevant insights.

**Table 4:**
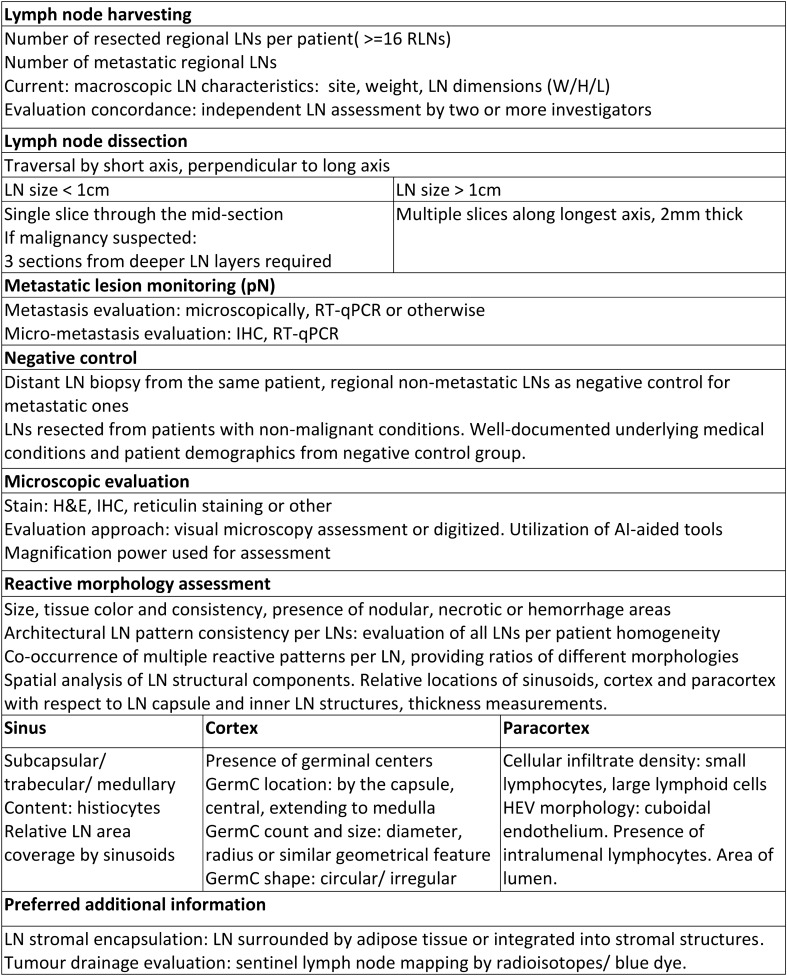
Recommended protocol for reporting lymph node morphology

*LN* lymph node, *RLN* regional lymph nodes, *IHC* immunohistochemistry, *RT-qPCR* reverse transcription quantitative polymerase chain reaction, *AI* artificial intelligence, *HEV* High endothelial venule, *GermC* germinal center

## Limitations

Our review has some limitations such as results originate from articles published mostly more than 30 years ago. Due to their publication dates, limited information could be retrieved and individual patient data were not available. In some cases, information could only be retrieved in an indirect way, making it a potential source of rounding errors.

Additionally, for the meta-analysis, only 5 studies were found to have sufficiently large patient numbers. Second, most of the included studies originated from the East. This may limit the generalization of our results as well as translation to the patients from Western world due to the different histology and treatment protocols between East and West [74].

## Conclusion

Although there are the above highlighted limitations, we conclude that the LN microarchitecture seems to bear valuable and new prognostically relevant information in GaC and OeC patients. In order to unravel the biology underlying morphologic changes in GaC and OeC and to translate this into the clinic, assessment of LNs in a standardized way is needed. In future, the retrieved information about the host’s anti-tumour immune mechanisms might be used for personalized patient treatment decisions in clinical practice.

### Supplementary Information

Below is the link to the electronic supplementary material.

Supplementary file1 (ZIP 2378 KB)
