# Supplementary material for: The clinical importance of the host anti-tumour reaction patterns in regional tumour draining lymph nodes in patients with locally advanced resectable gastric cancer: a systematic review and meta-analysis
Source: Gastric Cancer. 2023 Sep 30;26(6):847–62. doi: 10.1007/s10120-023-01426-w (PMC10640417; doi:10.1007/s10120-023-01426-w)
Supplement: Supplementary file 1 — Supplementary file1 (ZIP 2378 KB) [file 10120_2023_1426_MOESM1_ESM.zip › Supplements_070923/Overview Supplementary.docx]

| Reaction patterns | Supplementary figure S1 |
| --- | --- |
| Forest plots | Supplementary figure S2A-S2O |
| Classification system by Cottier et al. | Supplementary table S1 |
| Search Strategy | Supplementary table S2 |
| QUIPS risk of bias score | Supplementary table S3 |
| Data extraction | Supplementary table S4 |
| IHC Studies | Supplementary table S5 |
