# Supplementary material for: The clinical importance of the host anti-tumour reaction patterns in regional tumour draining lymph nodes in patients with locally advanced resectable gastric cancer: a systematic review and meta-analysis
Source: Gastric Cancer. 2023 Sep 30;26(6):847–62. doi: 10.1007/s10120-023-01426-w (PMC10640417; doi:10.1007/s10120-023-01426-w)
Supplement: Supplementary file 1 — Supplementary file1 (ZIP 2378 KB) [file 10120_2023_1426_MOESM1_ESM.zip › Supplements_070923/Supplementary Figure S1 Reaction patterns.docx]

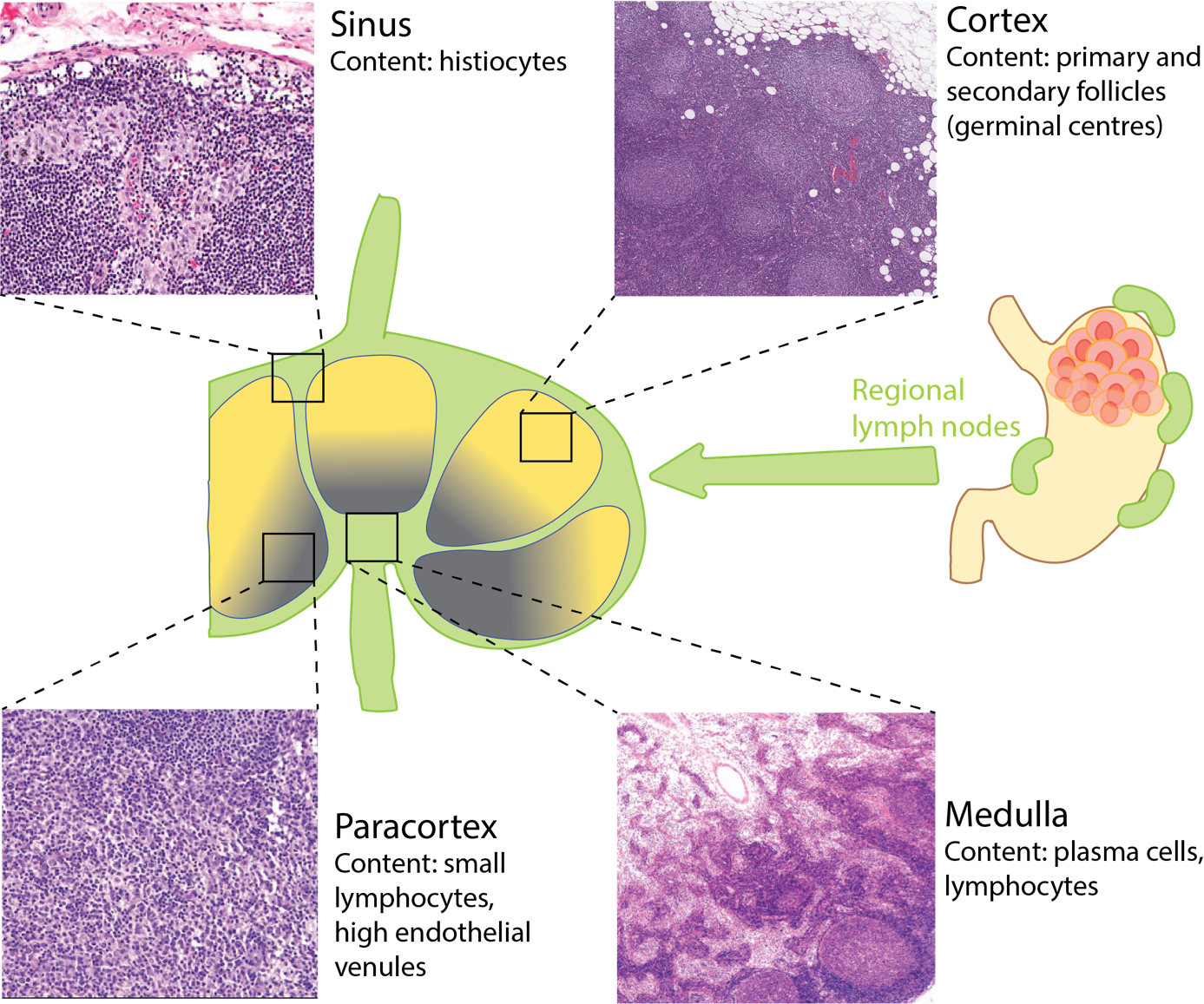


Supplementary Figure S1. Figure showing the reactive patterns in the lymph node (sinus hyperplasia (SH) in the sinusoids, follicular hyperplasia (FH) with germinal centers (GermC) and paracortical hyperplasia (PH) as evaluated in most of the studies. Medullary hyperplasia is also seen in lymph nodes but was not taken into account in the included studies.
