# Supplementary material for: The clinical importance of the host anti-tumour reaction patterns in regional tumour draining lymph nodes in patients with locally advanced resectable gastric cancer: a systematic review and meta-analysis
Source: Gastric Cancer. 2023 Sep 30;26(6):847–62. doi: 10.1007/s10120-023-01426-w (PMC10640417; doi:10.1007/s10120-023-01426-w)
Supplement: Supplementary file 1 — Supplementary file1 (ZIP 2378 KB) [file 10120_2023_1426_MOESM1_ESM.zip › Supplements_070923/Supplementary figures S2 ForestPlots.docx]

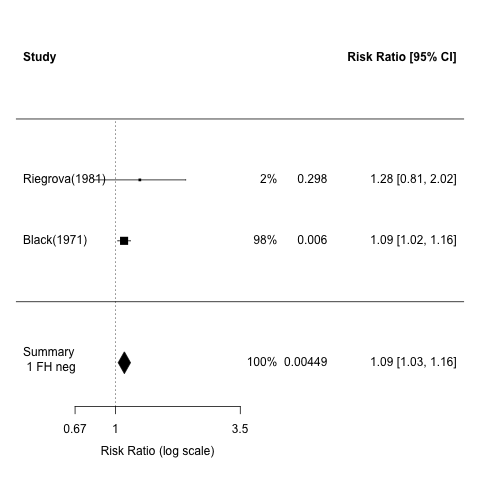


Figure S2A Forest plot depicting the meta-analysis of odds ratio estimates for 1 year overall survival (OS) for patients with follicular hyperplasia in non-metastatic lymph nodes.


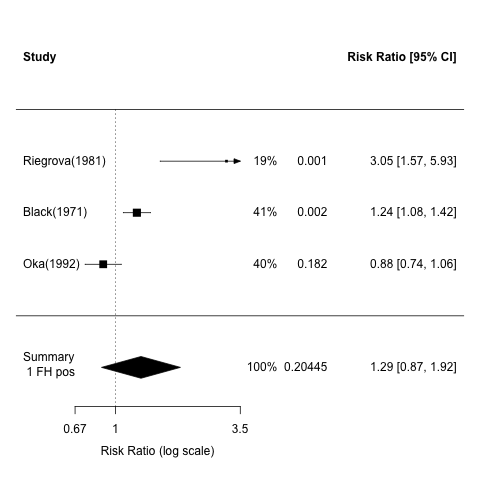


Figure S2B Forest plot depicting the meta-analysis of odds ratio estimates for 1 year overall survival (OS) for patients with follicular hyperplasia in metastatic lymph nodes.


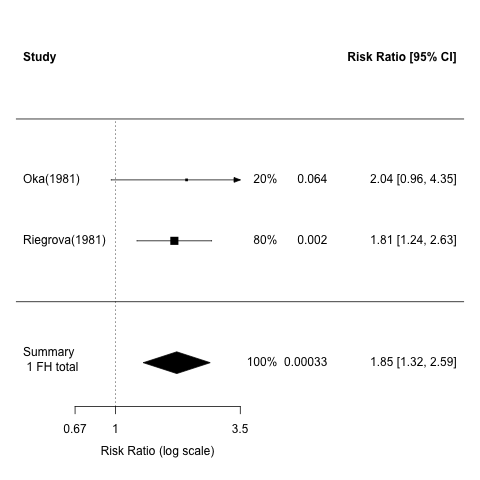


Figure S2C Forest plot depicting the meta-analysis of odds ratio estimates for 1 year overall survival (OS) for patients with follicular hyperplasia in lymph nodes irrespective of metastatic state.


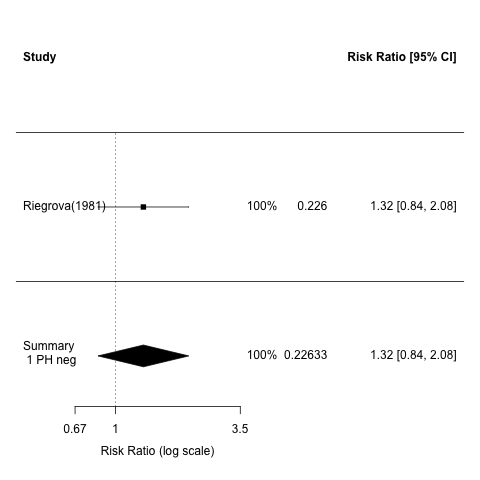


Figure S2D Forest plot depicting the meta-analysis of odds ratio estimates for 1 year overall survival (OS) for patients with paracortical hyperplasia in non-metastatic lymph nodes.


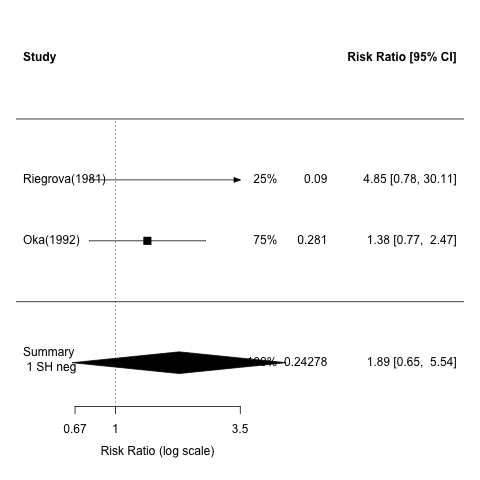


Figure S2E Forest plot depicting the meta-analysis of odds ratio estimates for 1 year overall survival (OS) for patients with sinus histiocytosis in non-metastatic lymph nodes.


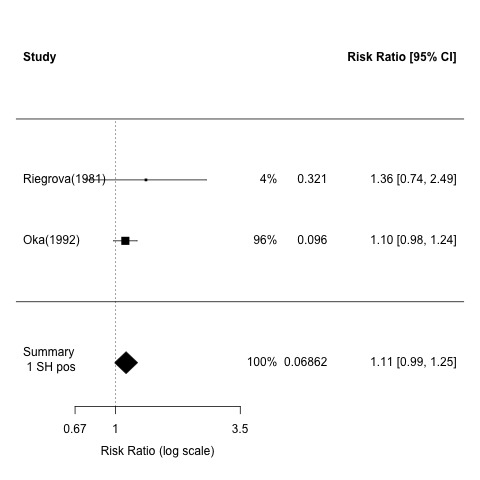


Figure S2F Forest plot depicting the meta-analysis of odds ratio estimates for 1 year overall survival (OS) for patients with sinus histiocytosis in metastatic lymph nodes.


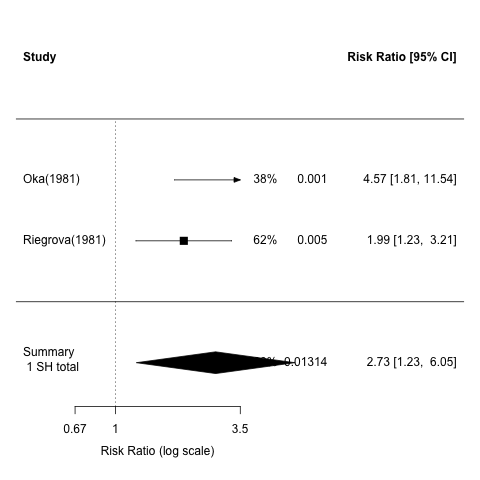


Figure S2G Forest plot depicting the meta-analysis of odds ratio estimates for 1 year overall survival (OS) for patients with sinus histiocytosis in lymph nodes irrespective of metastatic state.


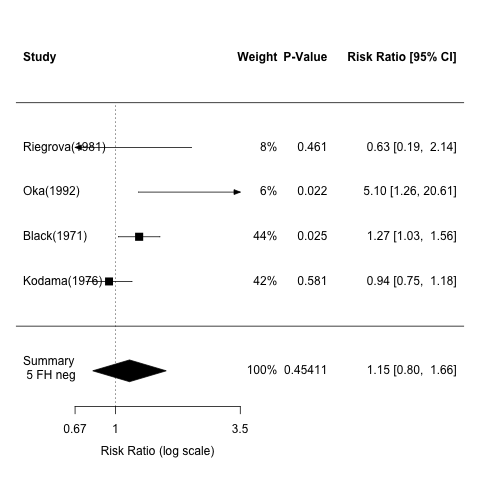


Figure S2H Forest plot depicting the meta-analysis of odds ratio estimates for 5 year overall survival (OS) for patients with follicular hyperplasia in non-metastatic lymph nodes.


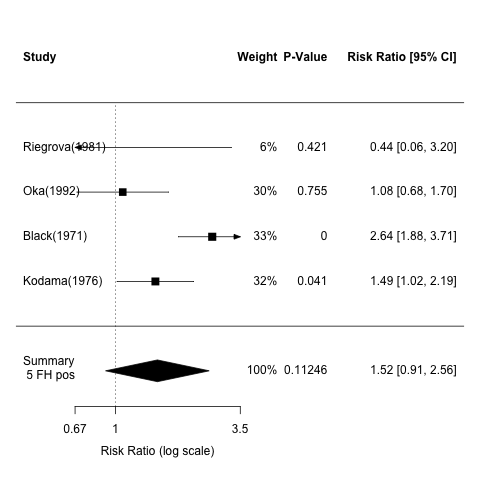


Figure S2I Forest plot depicting the meta-analysis of odds ratio estimates for 5 year overall survival (OS) for patients with follicular hyperplasia in metastatic lymph nodes.


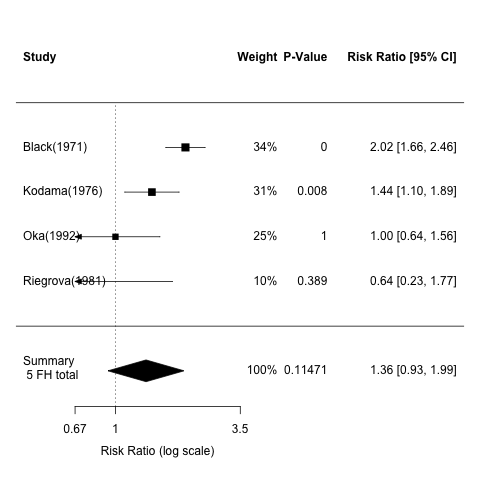


Figure S2J Forest plot depicting the meta-analysis of odds ratio estimates for 5 year overall survival (OS) for patients with follicular hyperplasia in lymph nodes irrespective of metastatic state.


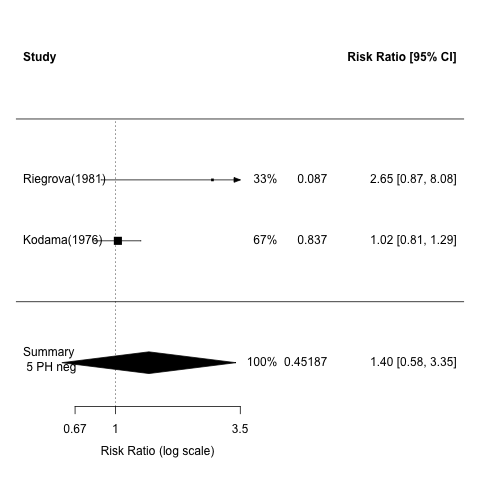


Figure S2K Forest plot depicting the meta-analysis of odds ratio estimates for 5 year overall survival (OS) for patients with paracortical hyperplasia in non-metastatic lymph nodes.


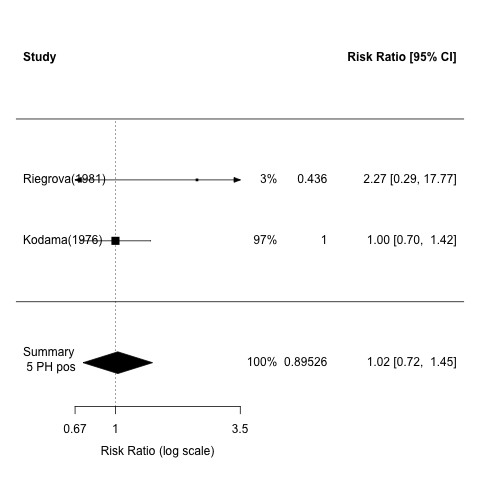


Figure S2L Forest plot depicting the meta-analysis of odds ratio estimates for 5 year overall survival (OS) for patients with paracortical hyperplasia in metastatic lymph nodes.


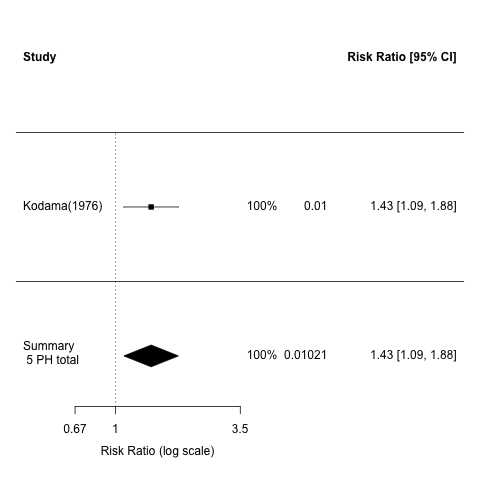


Figure S2M Forest plot depicting the meta-analysis of odds ratio estimates for 5 year overall survival (OS) for patients with paracortical hyperplasia in lymph nodes irrespective of metastatic state.


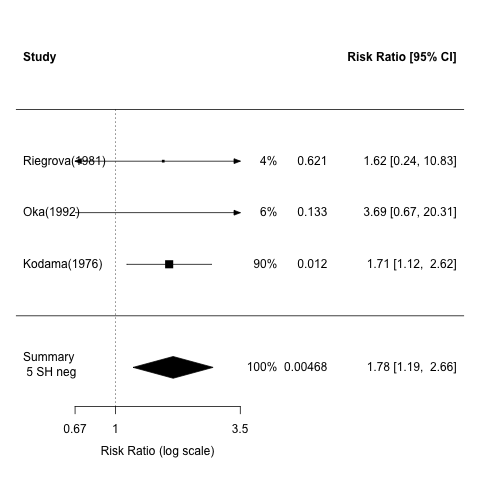


Figure S2N Forest plot depicting the meta-analysis of odds ratio estimates for 5 year overall survival (OS) for patients with sinus histiocytosis in non-metastatic lymph nodes.


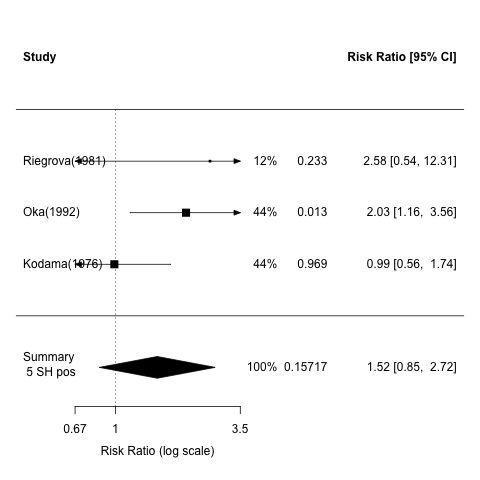


Figure S2O Forest plot depicting the meta-analysis of odds ratio estimates for 5 year overall survival (OS) for patients with sinus histiocytosis in metastatic lymph nodes.


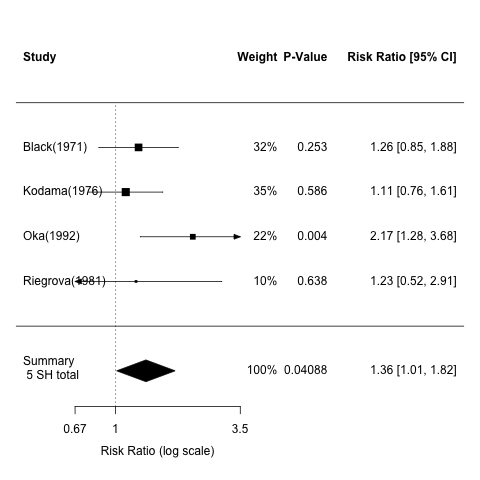


Figure S2P Forest plot depicting the meta-analysis of odds ratio estimates for 5 year overall survival (OS) for patients with sinus histiocytosis in lymph nodes irrespective of metastatic state.
