# Supplementary material for: The clinical importance of the host anti-tumour reaction patterns in regional tumour draining lymph nodes in patients with locally advanced resectable gastric cancer: a systematic review and meta-analysis
Source: Gastric Cancer. 2023 Sep 30;26(6):847–62. doi: 10.1007/s10120-023-01426-w (PMC10640417; doi:10.1007/s10120-023-01426-w)
Supplement: Supplementary file 1 — Supplementary file1 (ZIP 2378 KB) [file 10120_2023_1426_MOESM1_ESM.zip › Supplements_070923/Supplementary Table S1 Evaluation Cottier et al.docx]

Supplementary Table S1. Protocol for reporing of lymph node histology by Cottier et al.(1)


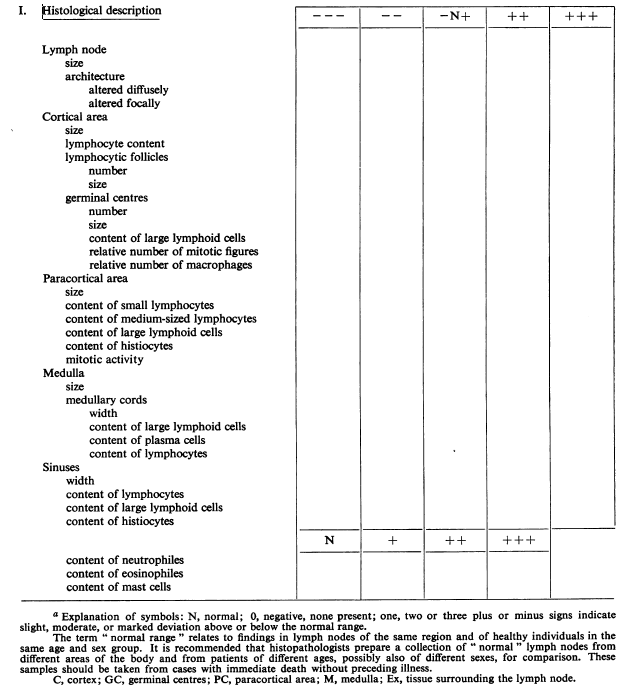


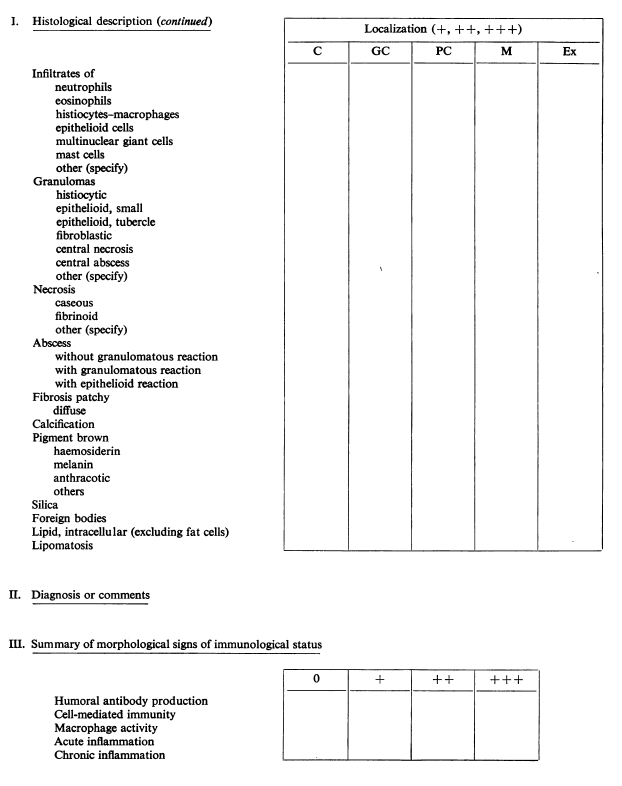

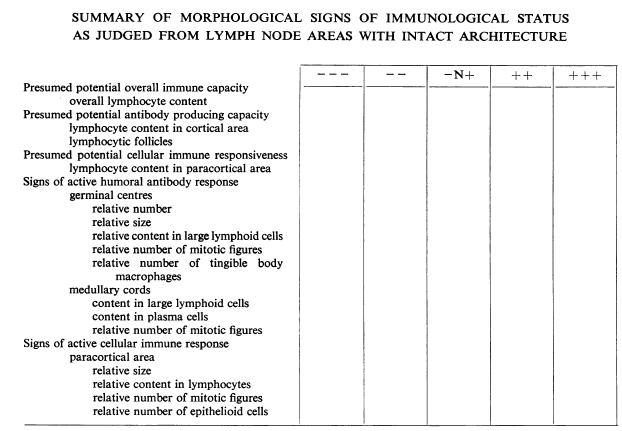


1. Cottier H, Turk J, Sobin L. A proposal for a standardized system of reporting human lymph node morphology in relation to immunological function. Bull World Health Organ. 1972;47(3):375-417.
