# Supplementary material for: The clinical importance of the host anti-tumour reaction patterns in regional tumour draining lymph nodes in patients with locally advanced resectable gastric cancer: a systematic review and meta-analysis
Source: Gastric Cancer. 2023 Sep 30;26(6):847–62. doi: 10.1007/s10120-023-01426-w (PMC10640417; doi:10.1007/s10120-023-01426-w)
Supplement: Supplementary file 1 — Supplementary file1 (ZIP 2378 KB) [file 10120_2023_1426_MOESM1_ESM.zip › Supplements_070923/Supplementary Table S2 Search strategies.docx]

Table S2. Search strategies with used search terms to build up search string.

| Patient population | Patients with oesophageal cancer, gastric cancer | Oesophageal Cancer  Gastric Cancer | (Neoplasm OR malignan* OR neoplasm* OR  tumor OR  tumour OR  cancer)  AND  exp esophagus/ OR  exp stomach/ OR  exp esophagus cancer/ OR  exp stomach cancer/ OR  esophag* OR  oesophag* OR  gastr* OR  stomach* OR  gastroesophag* OR  gastro-oesoph* ab,kw,ti. |
| --- | --- | --- | --- |
| Surrogate | Lymph Node | Lymph Node | exp lymph node/ OR  exp lymphoid tissue/ OR  exp lymph node dissection/  exp sentinel lymph node/ OR  exp sentinel lymph node biopsy/ OR  exp lymphatic system/ OR  Lymphoid tissue OR  Lymph node* OR  Lymph gland* OR  Lymphnode* OR  Sentinel node* OR  Metinel node* OR  Lymphatic system |
| Prognostic factor | Lymph Node reaction | Lymph node reaction pattern | exp Hyperplasia/ OR  exp Lymphocyte Activation/ OR  exp Pseudolymphoma/ OR  exp histiocytosis/ OR  exp sinus histiocytosis/ OR  exp germinal center/ OR  exp lymph follicle/ OR  exp lymphadenitis/ OR  exp granulomatosis/ OR  exp plasma cell/ OR  exp megakaryocyte/ OR  exp neutrophil/ OR  exp granulocyte/ OR  exp dendritic cell/ OR  exp Antigen-Presenting Cells/ OR  exp Phagocyte/ OR  lymph node hyperplasia OR  lymphoid hyperplasia OR  react* OR  Lymphadenopathy. OR  Histiocytosis OR  (germinal adj2 (center* or centre*)) OR  Lymph follicle OR  Lymphadenitis OR  Sarcoid like OR  Plasma cell* OR  Megakaryocyte* OR  Neutrophil* OR  Granulocyt* OR  Dendritic cell* OR  Sinus ectasia OR  (lymphocyte adj2 (predominance* or depletion*)) OR  (paracortical adj2 (hyperplasia* or activity*)) OR  (follicle adj2 (primary* or secondary* or tertiary)) OR  Tertiary lymphoid structures OR  (follicular adj2 (hyperplasia* or hypoplasia*)) OR  Dermatophatic OR  Granulomato* OR  Plasmocytosis OR  Immunophenotyping OR  Premetastatic niche |
| Outcome | Prognosis in terms of survival | Prognosis  Survival | exp survival/ OR  exp prognosis/ OR  exp treatment response/ OR  exp treatment outcome/ OR  exp survival analysis/ OR  Survival* OR  Prognos* OR  treatment outcome OR  treatment respons* OR  neoadjuvant* OR |
