# Supplementary material for: The clinical importance of the host anti-tumour reaction patterns in regional tumour draining lymph nodes in patients with locally advanced resectable gastric cancer: a systematic review and meta-analysis
Source: Gastric Cancer. 2023 Sep 30;26(6):847–62. doi: 10.1007/s10120-023-01426-w (PMC10640417; doi:10.1007/s10120-023-01426-w)
Supplement: Supplementary file 1 — Supplementary file1 (ZIP 2378 KB) [file 10120_2023_1426_MOESM1_ESM.zip › Supplements_070923/Supplementary Table S3 QUIPS.docx]

Supplementary Table S3. QUIPS analysis for bias risk assessment in reviewed studies.

| **Author(Year)** | **Study participation** | **Study attrition** | **Prognostic factor measurement** | **Outcome measurement** | **Study confounding** | **Statistical analysis and reporting** | **Overall risk bias** |
| --- | --- | --- | --- | --- | --- | --- | --- |
| Tokumoto(2014) |  |  |  |  |  |  |  |
| Ikeguchi(1998) |  |  |  |  |  |  |  |
| Takeya(2019) |  |  |  |  |  |  |  |
| Go(2016) |  |  |  |  |  |  |  |
| Jia(2015) |  |  |  |  |  |  |  |
| Otto(2014) |  |  |  |  |  |  |  |
| Kuriya(1979) |  |  |  |  |  |  |  |
| Oka(1981) |  |  |  |  |  |  |  |
| Lu(1993) |  |  |  |  |  |  |  |
| Syrjanen(1977) |  |  |  |  |  |  |  |
| Oka(1992) |  |  |  |  |  |  |  |
| Bedikian(1984) |  |  |  |  |  |  |  |
| Kojima(1980) |  |  |  |  |  |  |  |
| Lee(2011) |  |  |  |  |  |  |  |
| Kashimura(2011) |  |  |  |  |  |  |  |
| Takeya(2018) |  |  |  |  |  |  |  |
| Ishigami(2003) |  |  |  |  |  |  |  |
| Hiramatsu(2018) |  |  |  |  |  |  |  |
| O'Sullivan(1996) |  |  |  |  |  |  |  |
| Kloft(2021) |  |  |  |  |  |  |  |
| Okamura(1983) |  |  |  |  |  |  |  |
| Eriguchi(1984) |  |  |  |  |  |  |  |
| Black(1971) |  |  |  |  |  |  |  |
| Kodama(1976) |  |  |  |  |  |  |  |
| Riegrova(1981) |  |  |  |  |  |  |  |
| Donlon(2021) |  |  |  |  |  |  |  |
| Kumamoto(2021) |  |  |  |  |  |  |  |
| Itai(2021) |  |  |  |  |  |  |  |

| Legend | |
| --- | --- |
|  | Low bias risk |
|  | Medium bias risk |
|  | High bias risk |
|  | Not applicable |
