# Supplementary material for: The clinical importance of the host anti-tumour reaction patterns in regional tumour draining lymph nodes in patients with locally advanced resectable gastric cancer: a systematic review and meta-analysis
Source: Gastric Cancer. 2023 Sep 30;26(6):847–62. doi: 10.1007/s10120-023-01426-w (PMC10640417; doi:10.1007/s10120-023-01426-w)
Supplement: Supplementary file 1 — Supplementary file1 (ZIP 2378 KB) [file 10120_2023_1426_MOESM1_ESM.zip › Supplements_070923/Supplementary Table S4 Data Extraction Table.docx]

Suppementary Table S4. Data Extraction table.

| Category | Author(Year) | Type of cancer | Histology | Age mean/range | Sample size/ Male fraction (%) | Number of investigated LNs (* no data, one per patient inferred) | Neoadjuvant preoperative treatment | Type of staining | OS time | Evaluation method | Patient status or metastatic lymph node status | Research object | Main finding |
| --- | --- | --- | --- | --- | --- | --- | --- | --- | --- | --- | --- | --- | --- |
|  |  |  |  |  |  |  |  |  |  |  |  |  |  |
| LN compartment specific hyperplasias | Bedikian (1984) | Gastric | AC | 62/25-100 | 783/66.67 | 783* | 5-FU control group | H&E | 5 year | Qualitative |  | SH | SH associated with favorable outcomes |
|  | Kodama (1976) | Gastric | AC | -/- | 141/- | 2916 | None | H&E | 5 year | Qualitative |  | PH, SH, FH | PH, FH and PH FH combined associated with favorable outcomes, SH insignificant |
|  | Kuriya (1979) | Oesophageal | SCC | 63/47-71 | 21/- | 152 | not specified | H&E | 3 and 5 year | Qualitative |  | SH, FH | SH associated with favorable outcomes for 1-5 year survival, FH insignificant |
|  | Oka (1992) | Gastric | AC | -/32-81 | 102/61.77 | 3267 | None | H&E | 5 year | Qualitative |  | FH, FH | SH associated with favorable outcomes, FH insignificant |
|  | Okamura (1983) | Gastric | AC | -/- | 700/- | 3330 | not specified | H&E, methyl green pylonin stain | 5 year | Qualitative |  | SH, FH, PH | High rate of PH and FH in lymphoid stroma group. LN reaction pattern not related to survival |
|  | Black (1971) | Gastric | AC | -/- | 592/65.71 | 592* | None | H&E | 5 year | Qualitative |  | SH, FH, PH | FH associated with favorable outcomes |
|  | Kloft (2021) | Oesophageal | AC, SCC | 62.5/30-83.1 | 93/- | 93 | chemo+surgery | H&E | 5 year |  |  | LNneg size, lymphocytes, germinal centres, histiocytes density in LNs | Large LNneg have more germinal centres, less lymphocytes |
|  | Syrjanen (1977) | Gastric |  | -/- | 138/- | 295 | None | H&E | 5 year | Qualitative |  | PH, stromal lymphocyte reaction | PH associated with favorable outcomes |
|  | Eriguchi (1984) | Gastric |  | -/- | 33/- | 33* | BCG + chemoimmuntherapy | H&E | 2 year | Qualitative |  | SH | Group with BCG administration better OS, SH not related to OS |
|  | Oka (1981) | Oesophageal |  | -/- | 66/- | 66* | radiotherapy | H&E | 1 year | Qualitative |  | PH, FH, SH | SH associated with favorable outcomes in both radio and nonradio pretreatment groups, FH – only in no pretreated group |
|  | Kojima (1980) | Gastric, colon | AC | 51.5/32-68 | 32/- | 70 | not specified | H&E, methyl green pylonin stain | - | Qualitative |  | PH, FH, lymphocyte depletion, unstimulated | PH and FH associated to immune responsiveness |
|  | Lu (1993) | Oesophageal | SCC | -/19-72 | 1033/70.38 | 1033* | not specified | H&E | 10 year | Qualitative |  | SH, FH | FH more frequent in older age group, SH - no significant differnece |
|  | Riegrova (1981) | Gastric, breast | AC | -/36-80 | 80/60 | 80* | None | H&E | 5 year | Qualitative |  | PH, FH, SH, Lymphocyte depletion, unstimulated | PH and SH favorable prognostic indicators |
|  |  |  |  |  |  |  |  |  |  |  |  |  |  |
| Cell-type infiltration | Tokumoto (2014) | Gastric | AC | -/- | 52/- | 364 | None | H&E/IHC | 5 year | Manual cell counting per area | LNNeg vs micrometastatic LNs | TAN infiltration in LN compartments | TANs associated with unfavorable outcomes |
|  | Hiramatsu (2018) | Gastric |  | -/- | 120/72.5 | 480 | not specified | H&E/IHC | 5 year | Cell count via flow cytometry | LNM(-) LNNegs vs LNM(+) LNPos | CD15+TAN infiltration patterns in LNs | TANs associated with unfavorable outcomes |
|  | Takeya (2019) | Oesophageal | - | -/- | 182/87.91 | 182* | not specified | H&E/IHC | 10 year | 4 stage score by stained area percentage | LNM(-) LNNegs vs LNM(+) LNNegs | Maf-expressing macrophage densities in LNs | Gene expression heterogeneity in CD169+ LySMs |
|  | Go (2016) | Gastric | - | -/- | 49/- | 1448 | not specified | H&E/IHC | - | Manual cell counting per area | LNPos vs LNNeg | TAM spatial infiltration patters in LNs | CD163+ TAMs associated with unfavorable outcomes |
|  | Ishigami (2003) | Gastric |  | 60/31-86 | 27/62.96 | 35 | None | H&E/IHC | - | Automated cell counting, stained vs total cell ratio | SLN vs non-SLN, LNpos vs.LNNegs | S-100+ dendritic cell, T-cell, Mib-1+ cell infiltration in LNs | Immune cell spatial infiltrate signatures |
|  | Lee (2011) | Gastric | AC | -/- | 64/54.69 | 64* | None | H&E/IHC | - | Automated cell counting per area | LNM(-) and LNM(+), SLN positive vs non-SLN positive | Cell type diffusion patterns: cytotoxic Tcells, Tregs, NKs, DCs. | FoxP3+ Tregs associated with unfavorable outcomes |
|  | Takeya (2018) | Oesophageal | - | 65/- | 182/87.91 | 182* | chemo+chemoradiotherapy | IHC | 10 year | 4 stage score by stained area percentage | LNM(-) LNNegs vs LNM(+) LNNegs | CD169+ density in LNs | CD169+ LyMSs corelates with TIL count in primary tumour |
|  | Ikeguchi (1998) | Oesophageal | SCC | 64/- | 88/87.5 | 88* | None | H&E/IHC | 3 year | Manual cell counting per area | LNPos from LNM(+) vs LNNeg from LNM(-) | DC density in LNs | S100+ DCs associated with favorable outcomes |
|  | Kashimura (2011) | Gastric |  | 66.9/- | 123/72.36 | 123* | None | H&E/IHC | 10 year | Manual cell counting per area | LNM(-) and LNM(+) cases, LNNegs vs LNPos in LNM(+) cases | CD83 DC and Foxp3 Tregs density in LNs | CD83+ DCs associated with favorable outcomes, FoxP3+ Tregs – with unfavorable |
|  |  |  |  |  |  |  |  |  |  |  |  |  |  |
| Gene expression patterns | Otto (2014) | Oesophageal | - | -/- | 22/- | 44 | None | H&E/IHC | 2 year | Quantitative for DKK1-stained cells, and RT-PCR from frozen LN RNA samples | pN0 LNNegs vs pN1 LNNegs | DKK1-expressing cell distribution in subcapsular sinus, intermediary sinus and trabeculae od LNs | Downregulated DKK1 (Wnt inhibitor) expression in premetastatic TDLNs |
|  | O'Sullivan (1996) | Oesophageal | SCC | -/41-88 | 23/47.83 | 138 | None | None | 5 year | Quantitative optical density-based cytotoxicity assay in LN-derived lymphocyte cell culture | LNNegs in ESCC vs LNNegs in EAC | LN-derived lymphocyte reactivity differences between ESCC and AC | Immunosuppressed TDLNs in ESCC compared to adenocarcinoma control TDLNs |
|  | Jia (2015) | Oesophageal | SCC | 54/29-73 | 196/67.35 | 196* | None | H&E/IHC | 10 year | Semi-quantitative IHC-stained cell percentage and intensity scores, quantitative RT-PCR for frozen LN RNA samples | LN vs para-carcinoma tissue | Cell density difference evaluation in TDLNS with differentiable IDO expression | Low Bin1 and high IDO expression as independent prognostic negative factor |

Abbreviations: LN lymph node, OS overall survival, FU fluorouracil, BCG bacillus Calmette Guerin, H&E: hematoxylin and eosin, IHC immunohistochemistry, AC adenocarcinoma, SCC squamous cell carcinoma, SH sinus histiocytosis, PH paracortical hyperplasia FH follicular hyperplasia, LNneg tumour negative lymph node, LNpos tumour positive lymph node,,LNM lymph node metastasis, TAN tumour associated neutrophils, LySM: lymph node sinus macrophages, DC dendritic cells, TAM: tumour associated macrophages, DC dendritic cell, IDO indoleamine 2, 3-dioxigenase, DKK1 Dickkopf-related protein 1, TDLN tumour draining lymph node, CSS cancer-specific survival, RFS recurrence-free survival.
