# Supplementary material for: The clinical importance of the host anti-tumour reaction patterns in regional tumour draining lymph nodes in patients with locally advanced resectable gastric cancer: a systematic review and meta-analysis
Source: Gastric Cancer. 2023 Sep 30;26(6):847–62. doi: 10.1007/s10120-023-01426-w (PMC10640417; doi:10.1007/s10120-023-01426-w)
Supplement: Supplementary file 1 — Supplementary file1 (ZIP 2378 KB) [file 10120_2023_1426_MOESM1_ESM.zip › Supplements_070923/Supplementary Table S5 IHC studies.docx]

Supplementary Table S5. Summary of immunohistochemical assay studies on cellular composition changes in regional lymph nodes in patients of gastric or esophageal cancer.

| Cell type | Marker | Cancer Type | Link to Prognosis OS (p-value) | Clinical variable | Spatial distribution | Reference |
| --- | --- | --- | --- | --- | --- | --- |
| Dendritic cells | CD83+ | Gastric | Positive (p<0.0068) | ↑↓ TNM stage  ↑↓ LNmet  ↑↓ lymphatic and blood vessel involvement  ↑ histological differentiation | - | (1) |
|  | S-100+ | Esophageal | Positive(p=0.008), *in stages I/II vs III/IV** | ↑↓ LNmet  ↑ tumours with overexpressed mutated p53 | - | (2) |
|  |  | Gastric | N.A. | N.S. | GC | (3) |
|  |  |  | N.A. | ↑ distant LNmet |  | (4) |
|  | CD208+ | Gastric | N.A. | ↑↓ TNM stage  ↑↓ LNmet | P | (5) |
|  |  |  |  |  |  |  |
| Macrophages | CD169+ | oesophageal | Positive | ↑ TIL count in primary tumour after neoadjuvant treatment | S | (6) |
|  | Maf+CD169+ | oesophageal | N.S.** | N.S. | F and P | (7) |
|  | Maf+CD204+ | oesophageal | N.S.** | N.S. | - | (7) |
| Tumour-associated macrophages | CD163+ | Gastric | Negative | ↑ TNM stage  ↑ LNmet  ↑ disease recurrence  ↑ distant LN infiltration in presence of RLNmet | S (Marginal, intermedullary, medullary), MMs | (8) |
| Tregs | FOXP3+ | Gastric | Negative (p=0.01) | ↑ TNM stage  ↑ LNmet  ↑ lymphatic and blood vessel invasion | - | (1) |
|  |  |  | N.A. | ↑ TNM stage  ↑ LNmet | P and less in F | (5) |
| T cells | CD57+ | Gastric | N.A. | ↑↓ histological differentiation  ↑ LNmet | P, GC | (5) |
|  | CD8+ | Gastric | N.A. | ↑↓ histological differentiation | P | (5) |
|  | CD3-ζ+ | Gastric | N.A. | ↑↓ TNM stage | P | (3) |
| Tumor-associated neutrophils | CD15+ | Gastric | Negative (p=0.0039) | ↑ LNmet  ↑ Lymphatic vessel invasion  ↑↓ histological differentiation | S, around MMs | (9) |
|  |  |  | Negative (p=0.014) | ↑ TNM stage  ↑ LNmet | Around MMs, diffusely | (10) |
| Natural Killer cells | CD57+ | Gastric | N.A. | N.S. | - | (3) |
|  |  |  | N.A. | ↑↓ histological differentiation  ↑ LNmet | P, GC | (5) |
| Proliferation | MIB-1+ | Gastric | N.A. | ↑↓ depth of invasion | GC | (3) |

Symbols and abbreviations: ↑, ↑↓ - positive or negative correlation between cell density and state of the disease, N.S. – not significant, N.A. – not assessed, LNmet – metastatic lymph node, RLNmet – metastatic regional lymph node, ITCs – isolated tumour cells, LVD – lymphatic vessel density, lymph node compartments: S – sinuses, F – follicles, GC – germinal center, P – paracortex, MMs – micrometastases.

* 3-year OS

**10-year OS

1. Kashimura S, Saze Z, Terashima M, Soeta N, Ohtani S, Osuka F, et al. CD83(+) dendritic cells and Foxp3(+) regulatory T cells in primary lesions and regional lymph nodes are inversely correlated with prognosis of gastric cancer. Gastric cancer : official journal of the International Gastric Cancer Association and the Japanese Gastric Cancer Association. 2012;15(2):144-53.

2. Ikeguchi M, Ikeda M, Tatebe S, Maeta M, Kaibara N. Clinical significance of dendritic cell infiltration in esophageal squamous cell carcinoma. Oncology reports. 1998;5(5):1185-9.

3. S. I, S. N, Y. U, Y. H, A. N, F. M, et al. Infiltration of antitumor immunocytes into the sentinel node in gastric cancer. Journal of Gastrointestinal Surgery. 2003;7(6):735-9.

4. Tsujitani Si, Oka A, Kondo A, Ikeguchi M, Maeta M, Kaibara N. Infiltration of dendritic cells into regional lymph nodes in gastric cancer. Cancer. 1995;75.

5. Lee HE, Park DJ, Kim WH, Kim HH, Lee HS. High FOXP3+ regulatory T-cell density in the sentinel lymph node is associated with downstream non-sentinel lymph-node metastasis in gastric cancer. British journal of cancer. 2011;105(3):413-9.

6. Takeya H, Shiota T, Yagi T, Ohnishi K, Baba Y, Miyasato Y, et al. High CD169 expression in lymph node macrophages predicts a favorable clinical course in patients with esophageal cancer. Pathology international. 2018;68(12):685-93.

7. Takeya H, Ohnishi K, Shiota T, Saito Y, Fujiwara Y, Yagi T, et al. Maf expression in human macrophages and lymph node sinus macrophages in patients with esophageal cancer. Journal of clinical and experimental hematopathology : JCEH. 2019;59(3):112-8.

8. Go Y, Tanaka H, Tokumoto M, Sakurai K, Toyokawa T, Kubo N, et al. Tumor-Associated Macrophages Extend Along Lymphatic Flow in the Pre-metastatic Lymph Nodes of Human Gastric Cancer. Annals of surgical oncology. 2016;23:S230-5.

9. Tokumoto M, Tanaka H, Ohira M, Go Y, Okita Y, Sakurai K, et al. A positive correlation between neutrophils in regional lymph nodes and progression of gastric cancer. Anticancer research. 2014;34(12):7129-36.

10. Hiramatsu S, Tanaka H, Nishimura J, Sakimura C, Tamura T, Toyokawa T, et al. Neutrophils in primary gastric tumors are correlated with neutrophil infiltration in tumor-draining lymph nodes and the systemic inflammatory response. BMC immunology. 2018;19(1):13.
